# Fluoroquinolone Analogs, SAR Analysis, and the Antimicrobial Evaluation of 7-Benzimidazol-1-yl-fluoroquinolone in In Vitro, In Silico, and In Vivo Models

**DOI:** 10.3390/molecules28166018

**Published:** 2023-08-11

**Authors:** Mitzzy Fátima Medellín-Luna, Hiram Hernández-López, Julio Enrique Castañeda-Delgado, Fidel Martinez-Gutierrez, Edgar Lara-Ramírez, Joan Jair Espinoza-Rodríguez, Salvador García-Cruz, Diana Patricia Portales-Pérez, Alberto Rafael Cervantes-Villagrana

**Affiliations:** 1 Ciencias Farmacobiológicas, Facultad de Ciencias Químicas, Universidad Autónoma de San Luís Potosí, San Luis Potosí 78210, Mexico; mitzzy_medo@hotmail.com (M.F.M.-L.);; 2 Unidad Académica de Ciencias Químicas, Universidad Autónoma de Zacatecas, Zacatecas 98160, Mexico; 3 Unidad de Investigación Biomédica de Zacatecas, Instituto Mexicano del Seguro Social, Zacatecas 98000, Mexico; 4 Investigadores por México, CONAHCYT, Consejo Nacional de Humanidades, Ciencias y Tecnologias, Ciudad de México 03940, Mexico; 5 Centro de Investigación en Ciencias de la Salud y Biomedicina, UASLP, Sierra Leona No. 550, Lomas, San Luis Potosí 28210, Mexico; 6 Laboratorio de Biotecnología Farmacéutica, Centro de Biotecnología Genómica, Instituto Politécnico Nacional, Reyonsa 88710, Mexico; 7 Departamento de Cirugía Experimental e Investigación Quirúrgica y Bioterio, “Claude Bernard”, Área de Ciencias de la Salud, Universidad Autónoma de Zacatecas, Zacatecas 98160, Mexico

**Keywords:** structure–activity relationship, fluoroquinolone analog, antimicrobial, *S. aureus*

## Abstract

Structure–activity relationship (SAR) studies allow the evaluation of the relationship between structural chemical changes and biological activity. Fluoroquinolones have chemical characteristics that allow their structure to be modified and new analogs with different therapeutic properties to be generated. The objective of this research is to identify and select the C-7 heterocycle fluoroquinolone analog (**FQH 1–5**) with antibacterial activity similar to the reference fluoroquinolone through in vitro, in silico, and in vivo evaluations. First, SAR analysis was conducted on the **FQH 1–5**, using an in vitro antimicrobial sensibility model in order to select the best compound. Then, an in silico model mechanism of action analysis was carried out by molecular docking. The non-bacterial cell cytotoxicity was evaluated, and finally, the antimicrobial potential was determined by an in vivo model of topical infection in mice. The results showed antimicrobial differences between the **FQH 1–5** and Gram-positive and Gram-negative bacteria, identifying the 7-benzimidazol-1-yl-fluoroquinolone (**FQH-2**) as the most active against *S. aureus*. Suggesting the same mechanism of action as the other fluoroquinolones; no cytotoxic effects on non-bacterial cells were found. **FQH-2** was demonstrated to decrease the amount of bacteria in infected wound tissue.

## 1. Introduction

Over the last few decades, the World Health Organization (WHO) has considered antimicrobial resistance (AMR) an urgent public health problem [1], establishing strategies for its remediation [2,3]. One of these strategies is the development of new antibiotics focused on microorganisms classified as critical, high, or medium risk [4]. An estimated 13.7 million deaths worldwide are related to infections with AMR strains, where *S. aureus* is the microorganism responsible for more than 1 million deaths, followed by *E. coli*, *S. pneumoniae*, *K. pneumoniae*, and *P. aeruginosa* with 500,000 deaths [5].

The structural design of new chemical molecules with possible antimicrobial effects is guided by the structure of existing antibiotics with the purpose of improving their pharmacodynamic and pharmacokinetic properties [6,7]. However, these molecules must be studied during the different experimental phases including synthesis, chemical characterization, and biological activity [8]. To do this, structure–activity relationship (SAR) evaluations [9] are carried out, such as computer-aided drug design (CADD) through molecular docking [10]. The advantage of these CAAD techniques is the time and cost reduction for the synthesis processes, biological evaluations, and research [11].

In different SAR studies, fluoroquinolones have described the correlation between the different positions within the quinolone ring and their biological effects [12], facilitating the design of new molecules and thus producing hybrids, derivatives, or analogs [13,14]. The following is a brief description of the SAR of the structure of fluoroquinolones. In the C-1 position, there is a nitrogen atom (N1), so the insertion of cyclic groups generally favors antimicrobial potency. The C-3 and C-4 positions make up the pharmacophore of the molecule since the carboxylic acid group at C-3 and the keto group at C-4 come together to form the metal–water–amino acid complex through hydrogen and salt bridges between the oxo groups of these positions and Mg^2+^ with the amino acids: Serine, aspartate, and glutamate form the irreversible adduct, known as the quinolone resistance determining region (QRDR). The fluorine atom is in position C-6, which improves the penetrability of quinolones through the wall and membrane of Gram-positive and Gram-negative bacteria [15,16].

The C-7 position is the most flexible site to introduce bulky molecules capable of modifying antimicrobial activity [15,17]. To maintain the antibiotic effect of fluoroquinolones, it is recommended that the substituents in this region form a carbon-nitrogen bond (C7-N) between the atoms. Within the groups that improve the interaction with the protein are the heterocycles of the aromatic or aliphatic type, of five or six atoms, with or without fused cycles that allow volume increase [15]. At present, the increased microbial susceptibility to both types of bacteria of fluoroquinolones with heterocycles such as pyrimidine, indole, or imidazole has been demonstrated [15,18].

Fluoroquinolones dependently and independently inhibit DNA topoisomerase IIA (Topo II) enzymes [19]. The dependent pathway is through the formation of the metal-water-amino acid complex previously mentioned; the independent pathway is through the formation of stacking bonds with DNA structures, although this is a hypothesis [20,21,22]. By forming the aforementioned interactions, the tension of the supercoiled DNA strands originated by helicase activity during replication is increased, generating SOS signals of bacterial death [19,20,21,22]. Topo II enzymes are present in Gram-positive and Gram-negative bacteria; however, the affinity of fluoroquinolones is different for each type of bacteria, that is, in Gram-negative bacteria, fluoroquinolones act on DNA gyrase, while for Gram-positive, on Topo IV [23]. The therapeutic target for both proteins is similar because they have the same tyrosine domain and magnesium ion (Mg^2+^). In addition, the amino acid sequence encoding this region must contain the amino acids QRDR (serine, aspartate, and glutamate) [22,23].

The aim of this study was to identify and select the fluoroquinolone analog with heterocycle in position C-7 (**FQH 1–5**) with antibiotic activity similar to the reference fluoroquinolone in evaluations in vitro, in silico, and in vivo. From a SAR analysis of the five **FQH 1–5** fluoroquinolone analogs obtained by chemical synthesis, an evaluation was carried out in vitro of antimicrobial sensitivity, through which the compound with antibiotic activity similar to that of the reference compound in Gram-positive and Gram-negative bacteria was identified; subsequently, its mechanism of action was predicted in a model in silico by molecular docking. Its cytotoxic effect on non-bacterial cells was then determined, and finally, its antimicrobial effect was evaluated in an in vivo model of topical infection in mice.

## 2. Results and Discussion

### 2.1. Synthesis and Chemical Evaluation

Fluoroquinolone analogs with heterocycles at the C-7 position (**FQH 1–5**) were synthesized from the hydrolysis of compounds (**FQB 1–5**). The introduction of a weak nucleophilic agent such as amino heterocycles such as uracil, benzimidazole, tetrahydro-carbazole, 1,4-dihydropyridine, and 5,5-diphenyl-hydantoin was only possible with the formation of the boron complex with fluoroquinolone, as described by Miranda-Sánchez et al. [24] (see Figure 4). The difference in the FITR among **FQB 1–5** and **FQH 1–5** shows the changes in the peaks that confirm the hydrolysis in C-3 of fluoroquinolone analogs **FQH 1–5** (Figure S1). However, as the active form of the fluoroquinolone is the carboxylic acid at the C-3 position, the removal of the difluoroboryl part was carried out through basic hydrolysis with the use of NaOH (2N), showing medium to good reaction yields. Only the **FQH-5** molecule underwent some modifications in the part of the amino heterocycle linked at C-7 of the quinolone due to the presence of ester groups. The TLC demonstrates the absence of impurities in the solid compound obtained from **FHQ 1–5** after hydrolyzing **FQB 1–5** compounds (Figure S3). The carboxylic acid transformations were observed and confirmed by ^1^H NMR in a basic medium such as NaOH (Figure S2).

In the present investigation, only the antimicrobial activity of the **FQH 1–5** hydrolyzed compounds was evaluated because previous trials predicted a better outlook than **FQB 1–5**. In addition, there is little evidence regarding boronated fluoroquinolone complexes as antimicrobials; therefore, their evaluation in the different experimental models of the work was ruled out.

### 2.2. Structure–Activity Relationship (SAR) of the Fluoroquinolone Analogs FQH 1–5 against Gram-Positive and Gram-Negative Reference Bacteria

The SAR of fluoroquinolone analogs **FQH 1–5** was determined based on the antimicrobial effect obtained from the MIC and the MBC evaluated against the reference Gram-positive strains *S. aureus* and *E. faecalis*, as well as the Gram-negative strain *E. coli* and the strain of the clinical isolate *K. pneumoniae*. Additionally, the ciprofloxacin (**CPX**) patent formulation was used as a reference control and for the standardization of the method used in this work, by which the standard values of the concentrations of MIC and MBC on each of the bacterial strains were defined. The MIC data obtained is close to the reference values reported by the CLSI for each bacterial strain: *S. aureus* (0.250 mg/mL vs. 0.125–0.5 mg/mL), *E. faecalis* (0.250 mg/mL vs. 2–0.250 mg/mL), *E. coli* (0.013 mg/mL vs. 0.016–0.004 mg/mL) and *K. pneumoniae* (0.5 mg/mL vs. <1 mg/mL), as shown in Table 1. Therefore, the method used to demonstrate antibacterial susceptibility to **FQH 1–5** is suitable for standard bacterial strains.

In Table 1, the MIC and MBC values of the reference compound are shown: Ciprofloxacin (CPX) and the **FQH 1–5** for the description of the SAR between the heterocycle changes in each structure and the microbial susceptibility in each reference strain. The results demonstrated differences in the antimicrobial sensitivity of the **FQH 1–5** analogs, where the bacterium *E. coli* maintains a marked susceptibility to each of the molecules, though concentrations close to those of **CPX** were not reached in any compound.

In the analysis of the SAR, the chemical structural variability of each amino heterocycle attached to C-7 of the fluoroquinolone was reviewed, and two groups were detected: (1) Those that present carbonyls in the heterocycle, such as **FQH-3, 4,** and **5**, which showed MIC values higher than the reference (**CPX**) (>128 μg/mL), mainly in Gram-positive bacteria; and (2) those with fused rings, such as **FQH-1** and **2**, where it was observed that the MIC values were very close to the standard used on Gram-negative bacteria as well as on Gram-positive strains, obtaining concentrations of 32 μg/mL and 0.5 μg/mL for **FQH-1** and **FQH-2**, respectively. It is possible that the differences in the resulting MIC values have a close relationship with the binding of the aromatic heterocycle at C-7 of the fluoroquinolone, providing greater stability in the molecule due to electron conjugation and inducing a shift in electron density towards the carboxylic acid and keto group at C-3 and C-4, respectively. As compared to the tetrahydro-carbazole heterocycles in **FQH-1** and benzimidazole in **FQH-2**, both presented a dominant susceptibility to inhibit Gram-negative strains, having MIC and MBC values lower than those of the compounds **FQH 3–5**. Moreover, the **FQH-2** showed MIC values similar to those of **CPX** against *S. aureus* (0.250 µg/mL vs. 0.500 µg/mL). For this reason, a possible relationship is assumed between the aromatic heterocycles and how “bulky” the substitution attached to C-7 of fluoroquinolones is to improve interaction with the enzyme for bacterial inhibition. It is proposed that the decrease in aromatic character could be directly proportional to the antimicrobial effect presented in fluoroquinolones, such as in the case of **FQH-1**, in which the antimicrobial susceptibility decreases, compared to **FQH-2**, which is very similar to the standard; however, it is necessary to carry out a detailed mechanistic evaluation to confirm this hypothesis, which is not the object of study for the present work.

SAR evaluations previously reported in the literature for fluoroquinolones describe the influence of amino heterocycles made up of five or more carbon atoms joined at C-7 of the quinolone and the importance of improving and expanding the antimicrobial effect of quinoline derivatives [12,18,25]. Based on the above and according to the bacterial inhibition effect observed in the present work, some insights could be inferred: The fused heterocycle presented a broad spectrum of bacterial inhibition, acting mainly on *S. aureus*; likewise, a correlation was observed between the increase in aromatic character and bacterial inhibition, as in the case of **FQH-2.** This description is a suggested proposal in accordance with the results of this work since at present this type of structure has not been described. On the other hand, benzimidazole linked to fluoroquinolones has been studied through quinolone hybrids in N-1 [26] and its antimicrobial activity in reference strains (similar to those evaluated here) and in wild strains. However, comparing the results of these hybrids and the analogs of this work, it is found that the binding of benzimidazole is favored in C-7 of the fluoroquinolone more than in N-1, due to the fact that the antimicrobial action is improved against *S. aureus*. In summary, the structural changes that influence the antimicrobial activity in heterocycles are defined according to aromaticity, where they can be organized from lowest to highest antibiotic effect according to their proximity to the reference values (**CPX**) in the following manner: **FQH-5 < FQH-3 < FQH-4 < FQH-1 < FQH-2**. In addition, the affinity for bacterial types, where the aromatics (**FQH-1** and **2**) had greater antimicrobial sensitivity in Gram-positive bacteria, identified **FQH-2** as the leading compound for having inhibitory effects similar to **CPX** in the bacterium *S. aureus*. Therefore, evaluating the molecular patterns of binding with the Topo II protein provides evidence regarding the possible mechanism of action.

### 2.3. In Silico Model by Molecular Docking to Confirm the Antimicrobial Activity of FQH 1–5 and the Mechanism of Action of FQH-2

As mentioned, CADD tools such as molecular docking [11] provide the opportunity to expand SAR analyses of the contacts between the chemical structure under study and the different protein components, such as amino acids or catalytic ions present in the active site or “pocket” [10,25]. Therefore, it is possible through molecular docking to interpret and predict whether ligand–receptor binding is feasible by means of stability calculations (scores) in terms of binding energies (expressed in terms of Gibbs free energy, ∆G) [27]. In SAR studies in which molecular docking is used to screen molecules as derivatives of fluoroquinolones, by selecting the chemical structures that present the best ∆G values, the MICs for each derivative were subsequently verified. Our results demonstrated that heterocycles larger than six atoms enhance the inhibitory activity of both Gram-positive and Gram-negative bacteria [18,25]. This is further supported by previous data from other groups where fluoroquinolone derivatives with heterocycle amine in C-7 and chlorine atom in C-8 (**FQP-30**) in vitro studies (MIC) showed enhanced antibiotic activity [28]. However, when bringing the results to the in silico plane by molecular docking, they were found to be discordant, because the orientation and pose were different from the reference [29]. The mechanistic details of such SAR analysis await experimental evidence.

**Figure 1:**
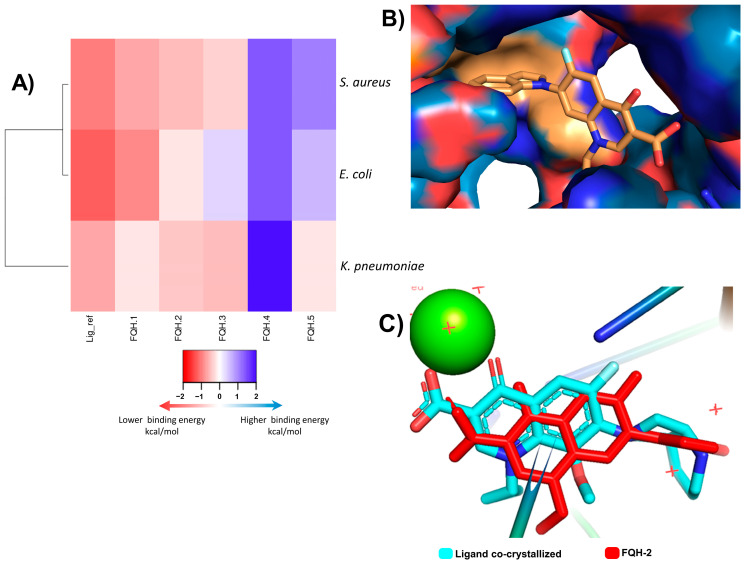
Molecular docking analysis of FQH-2 with topoisomerase II from several bacterial strains. Molecular docking was performed in a downloaded protein of PDB: *E. coli* (6rkv), *S. aureus* (5cdq), and *K. pneumoniae* (5eix), which included a co-crystallized fluoroquinolone, as well as a DNA and magnesium ion; the preparation of molecules was performed in the same way, except for the protein *E. coli,* in which all protein was taken as the pocket to direct the ligands and found inhibition allosteric sites and check the site to use the binding pocket. The docking process was performed with the program AutoDock Vina. The results were scored on binding energies analyzed in a heatmap, and the docked ligands were reviewed in the Pymol program. (**A**) Displays a heatmap on scores of binding energy; each cell represents a score of FQH 1–5 with Topo II, respectively. The colors indicate the strength of binding energies, where red represents lower energy (kcal/mol) and blue represents higher binding energy, the compounds with lower scores predict higher binding and therefore a plausible biological effect. The FQH-2 had lower scores for both bacterial strains (Gram-positive and Gram-negative). Therefore, it was selected for further analysis of the orientation and binding pose. (**B**) We show the interactions in the binding pocket through the topological area surface; orange color shows the contacts of FQH-2 with key amino acids of protein. (**C**) This picture shows the overlapping of moxifloxacin (cyan color) and FQH-2 (red color) in the target.

Among the applications of molecular docking is the prediction of antimicrobial activity, which could support the mechanism of action of these molecules derived from fluoroquinolones [30]. Molecular docking and simulation most likely cannot confirm the bioactivity results obtained experimentally. In contrast, experimental results may be able to confirm the modeling prediction. Therefore, in the present investigation, a screening model was proposed in silico focused on discerning those quinoline derivatives that project the greatest antimicrobial activity of fluoroquinolone analogs **FQH 1–5**. First, the pocket coordinates for each of the bacteria were corroborated by calculating the RMSD of the ligand-receptor model co-crystallized with Moxifloxacin (**MXF**) and the experimental molecular docking model. Our results show similar values between both models, which can be interpreted as precise coordinates for the location of the pocket. Furthermore, these binding energy score values are used as a reference in future comparisons with fluoroquinolone analogs **FQH 1–5**. In Figure 1, the results of the molecular docking of the **FQH 1–5** are shown with the different Topo II proteins from various bacterial strains showing the binding energy score through a heat map (see Figure 1A), where each cell represents the binding energy score value expressed in kcal/mol for each **FQH 1–5** and its respective Topo II protein. A red cell represents more negative energy values (greater ligand–receptor binding stability), while blue cells show lower ligand–receptor binding stability. Therefore, the results obtained from the calculated interactions between **FQH-1** and **FQH-2** with the different target proteins present uniformity in red cells with scores close to those of the reference. These results agree with the antimicrobial activity evaluated in the in vitro model, where **FQH-1** and **FQH-2** were the ones with the highest antimicrobial activity against Gram-positive and Gram-negative bacteria, as previously shown in Table 1.

Next, we sought to model the orientation of the **FQH-2** inside the protein pocket of *S. aureus* to propose a possible mechanism of action. The evaluation of the contact surface of the **FQH-2** in the DNA gyrase *S. aureus* (Figure 1B) shows a broad interaction with the cleavage site amino acids (orange color) such as ARG 122, GLY 459, GLU 585, and ASP 437, in addition to demonstrating that the ligand poses of the **FQH-2** and **MFX** (reference) are very similar (see Figure 1C). The slight differences found in the orientation between the **FHQ-2** and MXF are based on the heterocycle linked to C-7 of quinolone and are attributed to the property of planarity provided by benzimidazole with respect to the **MXF** bicycle and its influence on the final pose [18]. The heterocycle attached to the C-7 of Moxifloxacin is of the aza-bicycle type that has limited rotation at the sigma bond between the aza-bicycle nitrogen atom and the C-7 carbon in the fluoroquinolone, due to the steric hindrance provided by the methoxy at C-8 [31]. The **FQH-2** does not have substituents at C-8, allowing rotational torsion with more degrees of freedom where the preferred orientation of the benzimidazol-1-yl group is perpendicular to the plane of the quinolone. To summarize, a structural property that affects the pose or orientation of the molecule in the biological target is planarity, where having substituent groups with double bonds near the groups at C-7 will limit the rotation of the latter and hence their stability of interaction at the site of action.

As mentioned, the C-7 position in fluoroquinolone is key to modifying the structure and increasing the stability of the interaction with the protein. In the design of molecules **FQH 1–5**, changes in amino heterocycles in C-7 have shown differences in their biological activity. These are confirmed by the in silico model, where the results of the chosen compound **FQH-2** were very close to the reference (Moxifloxacin −11.3 kcal/mol vs. **FQH-2** −10.6 kcal/mol), a pattern resembling that of the in vitro results. This effect can be explained by the heterocycle (at C-7) characteristics, which allow the displacement of the electronic density on the functional groups at C-3 and C-4, substantially modifying the interactions with the amino acids in the “pockets”. Therefore, these results strongly suggest that the compound 7-benzimidazol-1-yl-fluoroquinolone is the optimal compound, in addition to proposing that **FQH-2** may present the antimicrobial mechanism of action by inhibiting Topo II; however, verification through biological assays is suggested. In addition, the evaluation of non-bacterial cells is necessary to rule out effects on Topo II proteins in general and to recognize the bacterial selectivity of the **FQH-2**.

### 2.4. Non-Cytotoxic Effect of FQH-2 on Non-Bacterial Cells by Flow Cytometry Assay

One of the pharmacodynamic properties of fluoroquinolones is that they act selectively against bacteria and other microorganisms rather than against human cells, due to the fact that even though prokaryotic (bacterial) and eukaryotic (human) Topo II DNA proteins are similar, the amino acid sequence of the catalytic (or cleavage) site is different [15], which suggests that the affinity of the fluoroquinolones is different and that they act preferentially in prokaryotic cells.

**Figure 2:**
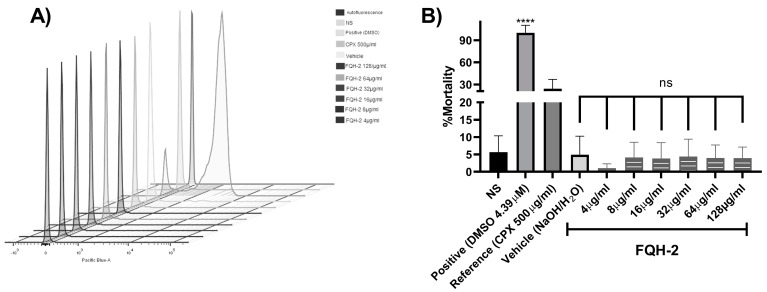
Non-cytotoxic effect of FQH-2 in peripheral blood mononuclear cells. PBMCs were extracted with lymphoprep and seeded at 500,000 cells per well in a 12−well plate. Non-treated (FBS 10%, also for all other conditions), positive (DMSO, 4.39 µM), CPX (ciprofloxacin, 500 mg/mL), vehicle (aqueous solution of H_2_O/NaOH), and increasing concentrations of 128−4 mg/mL of FQH-2. After 24 h of incubations, the cells were stained with kit live/dead staining reagent (Invitrogen), and lastly, each condition was acquired for flow cytometry; the data were analyzed in the FlowJo software (v.10.0). (**A**) We display the populations in a stacked histogram for each condition; the fluorescence increment (side right of x-axes) represents death population of cells, and the opposite is the viable population. (**B**) Percent mortality is shown based on flow cytometry data. No significant differences among FQH-2 concentrations were observed. The graph shows the mean standard deviation of three independent experiments. A One-way ANOVA statistical test with a Tukey post hoc test was performed with **** *p* < 0.0001.

Collectively, our results show that the molecule 7-benzimidazol-1-yl-fluoroquinolone (**FQH-2**) inhibits the Topo II bacterial enzyme; however, this enzyme is also present in non-bacterial cells. In addition, the benzimidazole molecule [32] has been shown to have antimicrobial activity [33], as well as antitumor [34], antiviral [35], and antifungal [36] activity. For this reason, the evaluation of cytotoxicity in non-bacterial cells is relevant. Consequently, it was necessary to determine if the **FQH-2** did not have toxic effects in eukaryotic or mammalian cells. To evaluate the cytotoxic effects of drugs or xenobiotics, it is necessary to use complex cells that may have a greater or constant exposure to them [37]; in this sense, peripheral blood mononuclear cells (PBMCs) are very useful cells for evaluating cytotoxic responses. In this work, the cytotoxic response of the mononuclear cells to **FQH-2** was evaluated by flow cytometry in a propidium iodide-based assay. We used a concentration range of 128 to 4 µg/mL (8-fold higher than the MIC obtained for *S. aureus*). The result of the distribution of the cell populations according to their condition can be observed in the histogram of Figure 2A, where the highest number of dead cells obtained were those exposed to DMSO (positive control), resulting in the highest fluorescence damage to the propidium iodide histogram, while for those exposed to **FQH-2,** the fluorescence amounts were lower, indicating low toxicity and the presence of viable cells. On the other hand, the mortality percentage data (Figure 2B) shows the non-cytotoxic effects of the different evaluated conditions, such as the one without stimulus (NS), which showed low levels of mortality (<5%). The cells exposed to CPX obtained a mortality < 20% higher than that of the NS control, while for **FQH-2** exposure, the mortality rates were lower than DMSO and CPX exposure for all concentrations.

Based on these results, the **FQH-2** demonstrates low cell toxicity, and we suggest that this may be due to its low affinity for Topo II DNA from non-bacterial cells. Based on reports of cytotoxic studies of the CPX (IC_50_) [38], the decrease in HeLa cell viability is demonstrated by Topo II DNA inhibition [39,40]. We show that despite using higher concentrations of the **FQH-2**, effects similar to those of the NS control were obtained, which indicates that **FQH-2** has no cytotoxic effect on blood cells in PBMCs and suggests a minimum risk of use in animal models.

### 2.5. Evaluation of the Antimicrobial Effect of FQH-2 in a Mouse Model of Topical Infection with S. aureus

According to the previous results, the **FQH-2** is the leading compound with the best antimicrobial activity against *Staphylococcus aureus*, which is an etiological and pathogenic agent of skin infections that could lead to a lethal disease such as impetigo [41,42]. Similarly, *S. aureus* is reported as the most common infectious agent in surgical site infections [43]. Therefore, we designed an *S. aureus* infection in surgical wounds to evaluate the antimicrobial effects of **FQH-2**. A hydrophilic base ointment containing **FQH-2** at 3% *w*/*w* was used. This had a homogeneous appearance of transparent color and colloidal consistency that favored easy application on contaminated surgical wounds. In addition, it was confirmed that this vehicle base did not influence bacterial inhibition or proliferation in comparison with other vehicle bases 

**Figure 3:**
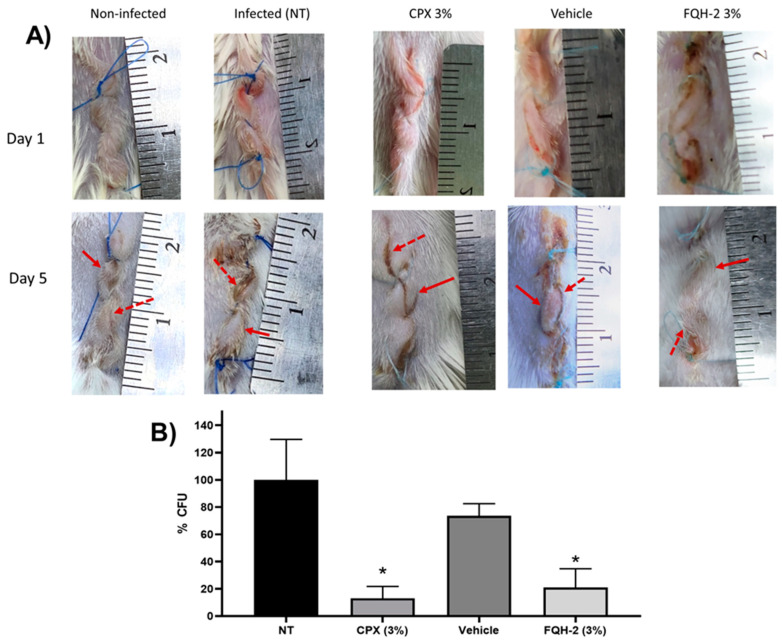
In vivo model of topical FQH-2 treatment and infection with *S. aureus* in mice. BALB/c mice were divided into five groups (*n* = 5): Non-infected (NT), infected, commercial ciprofloxacin ointment 3% (CPX 3%), vehicle (aqueous gel-based solution), and FQH-2 (formulation of FQH-2 in gel). Surgery and wound infection were performed on the animals according to the McRipley protocol. For the groups CPX, vehicle, and FQH-2, 5 h post-surgical and photographic documentation of the wounds was performed throughout the experiment. On day 5 the animals were sacrificed, and the tissues were collected and processed for CFU counts. (**A**) Representative images of a mouse for each group; the lines represent day 1 (surgery) and day 5 (end tracking), and the columns show the conditions. The arrows presented in the images refer to the main characteristics of wound infection, such as inflammation (continue arrow ↑) and wound edge swelling (discontinuous arrow⭫). (**B**) CFU counts were analyzed in wound tissues according to the infectious group. The comparison is made between the groups infected with treatment; hence, the CPX and FQH-2 had significant differences from the SE (infection) group, although the FQH-2 had a higher percentage than CPX. The graph shows the mean and standard deviation, statistical test of ANOVA one-way with Tukey post-test * *p* < 0.05.

The in vivo model of skin infections of surgical wounds in mice was shown to have the morphopathological features of contamination by *S. aureus*, causing physiological changes in the area associated with the infection. The physiopathological alterations are closely related to the inflammatory process, such as the formation of edema, reddening of the skin, and purulent discharge, as well as changes in the amount of bacteria present in the wound [44]. The experimental follow-up was five days later. In Figure 3A, representative images of the in vivo model are shown for topical infections of the experimental groups from day 1 (beginning) and day 5 (end). The first column contains the photos of the uninfected group, where a typical lesion pattern is observed on day 1; on day 5, the patterns of the lesion without bacterial contamination are observed, where no edema is seen throughout the tissue (identified with a continuous red arrow), just as the edges of the wound (discontinuous red arrow) are facing each other, which indicates that the correct healing process is taking place. Finally, no purulent secretions were observed, confirming that this group did not present an infection.

In the column of the infected group (without treatment: NT), the pattern of contaminated wounds is demonstrated in the photographs from the 5th day, where the redness of the skin and edema are observed (continuous arrow). In addition, purulent secretions are perceived between the edges of the wound (dashed arrow), which causes the edges to be separated, generating a delay in healing due to *S. aureus* infection. The subsequent columns are infected groups treated with **CPX** 3%, where the effect of the drug on the wound is observed since there is no presence of pus, the edges of the skin are facing each other, and while a small amount of edema is present, there is no erythema. All of these factors can be interpreted as the normal course of healing. The column of the group infected and treated with the formulation of **FQH-2** has a behavior similar to the non-infected control, showing moderate inflammation and erythema and apparent healing of the facing wound edges, unlike the group with the vehicle, whose last image is more similar to the pattern of the NT group. At this point, the images show a reduction of the infectious process with the administration of the **FQH-2**; however, it was necessary to confirm these results with the determination of the CFUs in the tissue of the contaminated wound.

In Figure 3B, the results for the amount of bacteria (% CFU) in the tissue of the infected wound are shown for each group (*n* = 5). The control groups of NT and vehicle had a similar bacterial burden since they did not present significant differences, meaning that the hydric ointment has no effect on bacterial proliferation or inhibition (*p* < 0.05). However, the control group NT and the antimicrobial formulations of **CPX** 3% and **FQH-2** showed a marked decrease in CFU: 13.08% ± 19.52 vs. 21.08 ± 27.38 respectively (*p* < 0.05). These data provide further evidence of the effective antimicrobial activity of **FQH-2.** Previous SAR studies on the antimicrobial activity of fluoroquinolones focused on studies in vitro, in silico, or cytotoxic; however, a further level of evaluation was reached in this work with the in vivo model in mice. The infection was carried out with the *S. aureus* bacterium due to its clinical importance as the main etiologic agent in various infections, causing millions of serious infections worldwide [45], in addition to being a bacterium with constant mutations that generate antimicrobial resistance [46]. Therefore, future evaluations are needed on the pharmacodynamic and pharmacokinetic properties of **FQH-2** and its effects on tissues.

## 3. Materials and Methods

### 3.1. Reagents

Ciprofloxacin (Senosiain^®^, Ciudad de México, México), dimethyl sulfoxide DMSO (Sigma-Aldrich, Saint Louis, MO, USA), culture medium as nutrient broth (BD Bioxon, Ciudad de México, Mexico), mannitol salt agar (BD Bioxon, Ciudad de México, México), tryptic soy agar (BD Difco™, Sparks, MD, USA), sodium chloride (J.T. Baker, Phillipsburg, NJ, USA), Triton X-100 (Sigma-Aldrich, Saint Louis, MO, USA), sterile 9% saline (AMSA Lab, Ciudad de México, Mexico), lymphoprep (STEMCELL, Vancouver, BC, Canada), LIVE/DEAD™ Fixable Dead Cells Staining Kits (Invitrogen, Carlsband, CA, USA), RPMI(Gibco™, Carlsband, CA, USA), Lymphoprep (Serumwerk, Bernburg, Germany), Sutures 4 zeros (American suture, Estado de México, México), sodium pentobarbital (Aranda Salud Animal, Guadalajara, Jalisco, México), ciprofloxacin ointment “Sophixin” (Sophia, Zapopan, Jalisco, México).

### 3.2. Instrumentation

Melting points were obtained using a Fisher-Johns melting point apparatus. IR spectra were performed on a Thermo Nicolet iS10 spectrophotometer using the attenuated total reflectance (ATR) technique. Nuclear magnetic resonance (NMR) spectra were obtained on a Bruker Ascend 400 MHz spectrophotometer, using DMSO-*d_6_* and TMS as internal standards.

### 3.3. Synthesis of Fluoroquinolone Analogs

**Figure 4:**
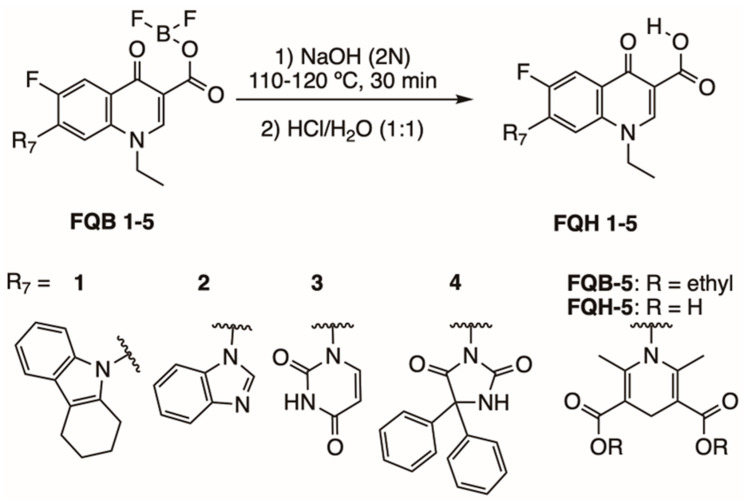
Synthesis of fluoroquinolone analogs **FQH 1–5** by basic hydrolysis from fluoroquinolone–boron complexes **FQB 1–5**.

Figure 4 shows the synthesis of fluoroquinolone analogs with heterocycles at position C-7 on **FQH 1–5** that was performed on the fluoroquinolone analogs **FQB-1** (7-(2,3,4,5-tetrahydro-carbazol-1-yl), **FQB-2** (7-benzimidazol-1-yl), **FQB-3** (7-uracil-1-yl), **FQB-4** (7-[5,5-diphenyl-hydantoin-1-yl]), and **FQB-5** (7-[3,5-diethoxycarbonyl-2,6-dimethyl-1,4-dihydropyridin-yl]), in the Organic Synthesis Laboratory of the Chemical Sciences Academic Unit of the Autonomous University of Zacatecas, Mexico, based on previous research focused on the synthesis of new fluoroquinolones with boron complexes [24].

Subsequent hydrolysis of the boron complex in fluoroquinolones **FQB 1–5** in basic medium to obtain the fluoroquinolone 3-carboxylic acid molecules **FQH-1** (7-carbazol-1-yl), **FQH-2** (7-benzimidazole-1-yl), **FQH-3** (7-uracil-1-yl), **FQH-4** (7-[5,5-diphenyl-hydantoin-1-yl]), and **FQH-5** (7-[2,6-dimethyl-1,4-dihydropyridine-3,5-dicarboxylic acid]) was carried out as follows:

A round-bottom flask was used with 100 mg of the boron complex of the fluoroquinolone (221.2 μmol for **FQB-1**; 250.6 μmol for **FQB-2**; 254.4 μmol for **FQB-3**; 187.5 μmol for **FQB-4**; or 187.1 μmol for **FQB-5**), and an aqueous solution of 2N NaOH (Sigma-Aldrich, Saint Louis, MO, USA) was added (792.6 μL for **FQB-1**; 898.2 μL for **FQB-2**; 911.9 μL for **FQB-3**; 672 μL for **FQB-4**; or 670 μL for **FQB-5**). The reaction mixture was refluxed for 30 min at a temperature of 110–120 °C and then slowly cooled to room temperature. An aqueous solution of HCl (Sigma-Aldrich, Saint Louis, MO, USA) (1:1) *v*/*v* was then added dropwise until pH 7 was reached. A white solid was obtained, which was vacuum filtered and washed with a neutral NaOH/HCl solution to remove excess NaOH. The corresponding 3-carboxylic acid-fluoroquinolone compounds **FQH 1–5** turned out with good yields. The **FQH 1–5** were dissolved in a 0.25 N NaOH aqueous solution and stored at room temperature.

#### Chemical Characterization of FQH 1–5

The molecule **FQH-1** (1-ethyl-7-(5*H*-1,2,3,4-tetrahydrocarbazole-5-yl)-6-fluoro-4-oxo-1,4-dihydroquinoline-3-carboxylic acid) is a white solid with a melting point (mp) of 318 °C and 73% (65.2 mg) of reaction yield. The FTIR-ATR, ν (cm^−1^): 3066–2946 (O–H, carboxylic acid), 1716 (C=O, carboxylic acid), 1616 (C=O, pyridone), 1561–1504 (C=C, aromatic), 1287–1212 (C–O, carboxylic acid), and 1093 (C–F, aromatic).

The 1-ethyl-7-(1*H*-benzimidazole-1-yl)-6-fluoro-4-oxo-1,4-dihydroquinoline-3-carboxylic acid labeled as **FQH-2** is a white solid with an mp of 330 °C and a 90% (79.2 mg) reaction yield. FTIR-ATR, ν (cm^−1^): 3208-2983 (O–H, carboxylic acid), 1704 (C=O, carboxylic acid), 1613 (C=O, pyridone), 1582–1542 (C=C, aromatic), 1299–1088 (C–O, carboxylic acid), and 1036 (C–F, aromatic).

The 1-ethyl-6-fluoro-4-oxo-7-(uracil-1-yl)-1,4-dihydroquinoline-3-carboxylic acid labeled **FQH-3** is a white solid with an mp of 311 °C and a 59% (51.8 mg) reaction yield. The FTIR-ATR, ν (cm^−1^) 3062-2926 (O–H, carboxylic acid), 1697 (C=O, carboxylic acid), 1615 (C=O, pyridone), 1558–1473 (C=C, aromatic), 1287-1229 (C–O, carboxylic acid), and 1042 (C–F, aromatic).

The 1-ethyl-6-fluoro-7-(5,5-diphenylhydantoin-3-yl)-4-oxo-1,4-dihydroquinoline-3-carboxylic acid labeled as **FQH-4** is a white solid with an mp of 297 °C and a 66% (60.1mg) reaction yield. FTIR-ATR, ν (cm^−1^): 3065–2948 (O–H, carboxylic acid), 1716 (C=O, carboxylic acid), 1616 (C=O, pyridone), 1560–1484 (C=C, aromatic), 1306–1093 (C–O, carboxylic acid), and 1041 (C–F, aromatic).

The 1-(3-carboxy-1-ethyl-6-fluoro-4-oxo-1,4-dihydroquinolin-7-yl)-2,6-dimethyl-1,4-dihydropyridine-3,5-dicarboxylic acid labeled as **FQH-5** is a white solid with an mp of 309 °C and a 50% (40.0 mg) reaction yield. FTIR-ATR, ν (cm^−1^): 3066–2961 (O–H, carboxylic acid), 1716 (C=O, carboxylic acid), 1616 (C=O, pyridone), 1561 (C=C, aromatic), 1213–1288 (C–O, carboxylic acid), and 1042–1093 (C–F, aromatic).

Thin-layer chromatography was performed to verify the absence of other synthetized products in the compound from dissolutions of **FQH 1–5** in aqueous media of H_2_O/NaOH 0.25 N using acetonitrile/ethanol (95:5) as the mobile phase at room temperature and a UV lamp at 254 nm.

### 3.4. Antimicrobial Activity

Bacterial strains. Four bacterial strains were evaluated in vitro, where two of them were the Gram-positive microorganisms *S. aureus* (ATCC 23235™) and *E. faecalis* (ATCC 29212™), and the other two were the Gram-negative *E. coli* (ATCC 25922™) and a strain of *K. pneumoniae* from a clinical isolate sensitive to ciprofloxacin. For the in vivo evaluation, a strain of *S. aureus* from a clinical isolate sensitive to ciprofloxacin and **FQH 1–5** was used. Both types of clinical isolates were obtained from the strain collection of the Experimental Immunotoxicology and Therapeutics Laboratory of the Academic Unit of Chemical Sciences at the Autonomous University of Zacatecas, Mexico.

### 3.5. In Vitro Antimicrobial Activity Assays for the Determination of the Minimum Inhibitory Concentration (MIC) and Minimum Bactericidal Concentration (MBC)

The review of the antibacterial activity of the compounds **FQH 1–5** was performed using the microdilution method in standardized broth, in accordance with the method reported by the Clinical and Laboratory Standards Institute (CLSI) M07 11th edition M100 30th [47,48]. The evaluation of the minimum inhibitory concentration (MIC) was carried out through microdilution in 96-well plates with 1.5 × 10^5^ CFU/mL of each of the bacteria mentioned in the previous section. Each one was exposed to a range of serial dilution concentrations according to the standardized values in the MIC break points of ciprofloxacin (**CPX**) (Senosiain^®^, Ciudad de México, México), specific for each bacterial strain, which also functioned as a reference control. **FQH 1–5** compounds were treated similarly with their respective concentrations for each bacterium. The same dilution conditions were used for the vehicle control, which consisted of an aqueous solution. All 96-well plates with the different conditions (**FQH 1–5**, **CPX,** and the vehicle) were incubated at 37 °C for 24 h until the experimental inhibitory concentration (breakpoint), which was determined when no turbidity was observed at the bottom of the well after 24 h of incubation.

The minimum bactericidal concentration (MBC) was determined by taking samples from the wells close to the MIC for each bacterial strain to later reseed them in Petri dishes with trypticase soy agar (BD Difco™,Sparks, MD, USA), and incubate them for 24 h at 37 °C. The value of MBC was established in areas where the development of colony-forming units (CFU) was not shown. All assays were carried out in triplicate for all bacterial strains and the **FQH 1–5** [48].

### 3.6. Molecular Docking

The in silico model was carried out by means of three crystallized proteins of DNA topoisomerase II (Topo II), downloaded from the Protein Data Bank (PDB) database shown in Table 2. These proteins have a co-crystallized fluoroquinolone in the active site [49]. The chemical structures of the **FQH 1–5** were drawn in the Marvin Sketch program (Marvin version 21.17.0, ChemAxon https://www.chemaxon.com, accessed on 12 September 2019). The proteins were edited to obtain the receptor and ligand in the USCF Chimera program [50]. The coupling of the ligand for each receptor in its respective bacterial cell was carried out through the AutoDock Vina program, which uses the Lamarckian genetic algorithm (LGA) and is based on semi-empirical free energy fields. In the AutoDock program, tools were used to identify the coordinates and sizes of the grid box to dock the **FQH 1–5**, based on the location of the co-crystallized ligand. Results are shown from the binding energy score, as the ligand pose was retained for analysis. Co-crystallized ligand docking scores were taken as the reference values [51] (used to compare the results of docked FQHs). All the proteins had the same treatment, with the exception of *E. coli* (6rkv). Due to its characteristics, a different molecular strategy was used, since it was performed through blind docking [52], where the complete structure of the protein was taken as the pocket (grid box), and sites of interaction were confirmed using the Protein Plus server [53]. In addition, interactions in this model were evaluated with the Pymol program [54]. For a better understanding of the binding energy scores, analysis was conducted through a heat map using the Heat Mapper server [55].

### 3.7. Cytotoxicity Assays

Venous blood obtained from healthy volunteers in EDTA tubes was diluted 1:1 with RPMI (Gibco™, Carlsband, CA, USA), medium and carried with Lymphoprep™ (Serumwerk, Bernburg, Germany) in a 15 mL propylene conical tube [59]. After centrifugation (800× *g*, 30 min), peripheral blood mononuclear cells (PBMCs) were seeded and stored in a conical tube with fresh RPMI medium, and cells were washed three times with phosphate-buffered saline (450× *g*, 5 min). Finally, the PBMCs were resuspended in RPMI medium supplemented with 10% fetal bovine serum (FBS), and cell number and viability (>95%) were determined. Next, 500,000 cells per well were seeded in a 12-well plate, and the cells were exposed to different conditions [60]: Without stimulation (viability control), at a concentration of 4.39 µm of dimethyl sulfoxide (DMSO) as a positive control, toxic concentrations of ciprofloxacin at 500 µg/mL (reference control), an aqueous solution of H_2_O/NaOH (vehicle control), and increasing concentrations of the selected **FQH 1–5**. Cells were incubated at 36 °C for 24 h with 5% CO_2_. Afterwards, cells in suspension were collected, transferred to polystyrene round-bottom tubes, and washed twice with PBS. Cell staining was then performed using the LIVE/DED™ Fixable Dead Cell Stain Kit dye (Invitrogen, Carlsband, CA, USA) following the manufacturer’s instructions. Finally, all the samples were measured in a FACSCanto II flow cytometer with a 4-2-2 configuration (Becton Dickson, Franklin Lake, NJ, USA) and analyzed with the FlowJo v 10.0 program (BD Bioscience, Franklin Lake, NJ, USA).

### 3.8. Evaluation of the Antimicrobial Activity of FQH-2 in an In Vivo Model of Topical Infection

*Animals.* Pathogen-free BALB/c mice with a weight range between 25 and 30 g were used in all experiments, purchased from a commercial source (Centro de Biociencias de la Universidad Autónoma de San Luis Potosí, San Luis Potosí, México). The animals were cared for in the Bioterio “Claude Bernard” in the Area of Health Sciences of the Autonomous University of Zacatecas, Zacatecas, Mexico. They were kept in individual boxes at a temperature of 22–24 °C with controlled humidity and were allowed food and water *ad libitum* [61]. The animals were divided into five groups (n = 5): A group without infection (non-infected), which were mice with surgical wounds without infectious agents. Groups of mice with *S. aureus*-infected wounds were treated as follows: no treatment (NT) group; 3% ciprofloxacin (**CPX**) (Sophixin™ ointment) (Sophia, Zapopan, Jalisco, México). group; vehicle group (aqueous solution ointment); and previously prepared 3% **FQH-2** ointment administration group.

*Ointment preparation.* An **FQH-2** ointment was prepared from a hydrophilic base: 50 mg of carbopol was completely dissolved in 10 mL of a solution of sterile distilled water and 3% **FQH-2**. One milliliter of triethanolamine was then added with constant stirring until the consistency changed to colloid. Finally, 3% **FQH-2** ointment (prepared from a premade 5 mg/mL solution) was stored at room temperature [62].

*In vivo model of topical infection*. The antimicrobial effect of the **FQH-2** was evaluated according to the McRipley and within protocol, with some material adaptations. In this topical infection model, suture contamination was performed with a strain of *S. aureus* (sensitive to ciprofloxacin). To do this, an inoculum of *S. aureus* (2 × 10^9^ CFU/mL) in a 0.85% NaCl solution was prepared, to which sterile medical sutures (11 cm) (American suture, Estado de México, México) were added for 30 min and then dried on a sterile surface [63,64,65,66]. Mice were anesthetized with 75 mg/kg pentobarbital (Aranda Salud Animal, Guadalajara, Jalisco, México), and topical lidocaine [67]. The lesion area was sterilized with aseptic solutions, and surgery was performed on the back of the shaved animals by means of a 2 cm longitudinal cut in the downward direction of the meaty panniculus under sterile conditions [66]. At the end of the procedure, the recovery of the animals was monitored. The infectious process was followed for five days. After this, the animals were sacrificed according to NOM-033-ZOO-1995 with CO_2_ in a pathogen-free environment. Wound tissue (uninfected and infected) was collected and processed for bacterial counting, which was performed after maceration of the tissue with a sterile solution of NaCl 0.85%. A serial dilution was then carried out. To take a sample of each of the dilutions and sow them in Petri dishes with salt and mannitol agar (BD Bioxon, Ciudad de México, México), they were incubated for 24 h at 37 °C. After this time, the colonies were counted (CFU/mL) in order to gather all the data for the analysis and interpretation of the results [62,63,64,66].

*Dosage.* The administrations were 500 mg of ointment in each animal, starting at 5 h post-surgery, with subsequent dosages carried out every 12 h until day 5. The application of each of the formulations was performed with a metal spatula (previously sterilized) and in an aseptic environment.

This methodology was approved by the Bioethics Committee of the Health Sciences Area of the Autonomous University of Zacatecas, with registration number ACS/UAZ182/2022. All procedures were performed in accordance with NOM-062-Z00-199, as well as under consideration of international guidelines for choosing the correct end point in research with experimental animals [61,65].

### 3.9. Statistical Analysis

The data from the experimental results were analyzed in the Graph Pad Prism version 8.0 program. Normality tests were performed to later apply multiple comparison tests between the groups using the one-way ANOVA test with Tukey’s post-test. Significant difference values were considered from * *p* < 0.05 to **** *p* < 0.0001 in all analyses.

## 4. Conclusions

From the SAR evaluation of fluoroquinolone analogs FQH 1–5, it was shown that fused aromatic heterocycles in position C-7 improve antimicrobial susceptibility in Gram-negative bacteria and mainly in Gram-positive ones. The FQH-2 compound (7-benzimidazole-1-yl-fluoroquinolone) was found to have the highest antimicrobial activity against *S. aureus*. In addition, during the antimicrobial evaluation using different experimental models, similar antimicrobial profiles and inhibitory activity among FQH-2 and ciprofloxacin were observed. However, one advantage of FQH-2 over CPX is its lower cytotoxicity against non-bacterial cells. For this reason, it was possible to show the antibiotic effect of FQH-2 in the topical infection model in animals, where a decrease in CFU of *S. aureus* bacteria was detected, which in turn contributed to the overall wound healing process. It is recommended that future research be directed towards the evaluation of resistant strains such as *S. aureus* to confirm its possible use against infections of strains with AMR and to further characterize the pharmacodynamic and pharmacokinetic properties of FQH-2.

## Figures and Tables

**Table 1:** MIC and MBC values of reference fluoroquinolone (CPX) and fluoroquinolone analogs **FQH 1–5**.

Label	Structure	*S. aureus* (ATCC 25923)		*E. faecalis* (ATCC 29212)		*E. coli* (ATCC 25922)		*K. pneumoniae* *	
		MIC (mg/mL)	MBC (µg/mL)	MIC (µg/mL)	MBC (µg/mL)	MIC (µg/mL)	MBC (µg/mL)	MIC (µg/mL)	MBC (µg/mL)
**CPX**	CLSI Values 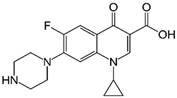 C_17_H_18_FN_3_O_3_ experimental values	0.125–0.5	0.250	0.250–2	0.250	0.016–0.004	0.008	<1	1
		0.250	0.250	0.250	0.250	0.013 ± 0.005 ^a^	0.033 ± 0.002 ^a^	0.5	0.5
**FQH-1**	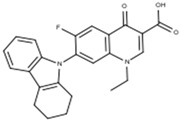 C_24_H_21_FN_2_O_3_	32	>128 ^b,c^	128 ^b^	>128 ^b,c^	1.667 ± 0.577 ^a^	4	64	64
**FQH-2**	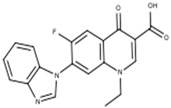 C_19_H_14_FN_3_O_3_	0.5	0.5	4	4	1.333 ± 0.577 ^a^	2	16	32
**FQH-3**	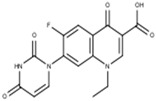 C_16_H_12_FN_3_O_5_	128	128	128	>128 ^b,c^	1.667 ± 0.577 ^a^	4	128	128
**FQH-4**	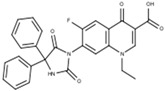 C_27_H_21_FN_3_O_5_	128	>128 ^c^	128	>128 ^b,c^	2	4	32	32
**FQH-5**	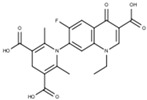 C_21_H_22_FN_2_O_7_	128	>128 ^b,c^	128	>128 ^b^	4	4	64	64

^a^ Variance caused by the scope of the technique with no statistically significant differences. ^b^ Values greater than the concentrations reached by the solubility in the aqueous medium NaOH/H_2_O. ^c^ Values without bactericidal effect. * Clinical isolate sensitive to fluoroquinolone.

**Table 2:** Protein characteristics.

Strain	PDB Code	DNA Topoisomerase IIA	Resolution (Å)	Adjunct Molecules	Reference
*Klebsiella pneumoniae*	5eix	DNA topoisomerase IV	3.35	Magnesium Levofloxacin	[56]
*Staphylococcus aureus*	5cdq	DNA gyrase	2.95	Glycerol Magnesium Moxifloxacin	[57]
*Escherichia coli*	6rkv	DNA gyrase	4.60	----	[58]

## Data Availability

Not applicable.

## Supplementary Materials

The following supporting information can be downloaded at: https://www.mdpi.com/article/10.3390/molecules28166018/s1.

## Sample Availability

Samples of the compounds are not available from the authors.
