# Supplementary material for: Fluoroquinolone Analogs, SAR Analysis, and the Antimicrobial Evaluation of 7-Benzimidazol-1-yl-fluoroquinolone in In Vitro, In Silico, and In Vivo Models
Source: Molecules. 2023 Aug 11;28(16):6018. doi: 10.3390/molecules28166018 (PMC10458221; doi:10.3390/molecules28166018)
Supplement: Supplementary file 1 [file molecules-28-06018-s001.zip › molecules-2531806-supplementary.pdf]

Supplementary Information

Fluoroquinolone Analogs, SAR Analysis, and the Antimicrobial Evaluation of 7-Benzimidazol-1-yl-Fluoroquinolone in In Vitro, In Silico, and In Vivo Models

Mitzzy Fátima Medellín-Luna [1,2,3], Hiram Hernández-López [2], Julio Enrique Castañeda-Delgado [3,4], Fidel Martinez-Gutierrez [1,5], Edgar Lara-Ramírez [6], Joan Jair Espinoza-Rodríguez [2], Salvador García-Cruz [7], Diana Patricia Portales-Pérez [1,5] and Alberto Rafael Cervantes-Villagrana [2,*]

1 Ciencias Farmacobiológicas, Facultad de Ciencias Químicas, Universidad Autónoma de San Luís Potosí, San Luis Potosí 78210, Mexico; mitzzy_medo@hotmail.com (M.F.M.-L.); fidelmicro@gmail.com (F.M.-G.); dportale@uaslp.mx (D.P.P.-P.)
2 Unidad Académica de Ciencias Químicas, Universidad Autónoma de Zacatecas, Zacatecas 98160, Mexico; hiram_hdez@hotmail.com (H.H.-L.); espinoza.rodriguez.jj@gmail.com (J.J.E.-R.)
3 Unidad de Investigación Biomédica de Zacatecas, Instituto Mexicano del Seguro Social, Zacatecas 98000, Mexico; julioenriquecastaneda@gmail.com
4 Investigadores por México, CONAHCYT, Consejo Nacional de Humanidades, Ciencias y Tecnologias, Ciudad de México 03940, Mexico
5 Centro de Investigación en Ciencias de la Salud y Biomedicina, UASLP, Sierra Leona No. 550, Lomas, San Luis Potosí 28210, Mexico
6 Laboratorio de Biotecnología Farmacéutica, Centro de Biotecnología Genómica, Instituto Politécnico Nacional, Reyonsa 88710, Mexico; elarar0700@hotmail.com
7 Departamento de Cirugía Experimental e Investigación Quirúrgica y Bioterio, "Claude Bernard", Área de Ciencias de la Salud, Universidad Autónoma de Zacatecas, Zacatecas 98160, Mexico; sgc_54@uaz.edu.mx
* Correspondence: dr.albertocervantes@uaz.edu.mx

Content

Binding energy score for docking calculations **S2**
FTIR – ATR spectra **S3**
NMR spectra **S5**
Thin Layer Chromatography **S6**

Binding energy score for docking calculations

**Table S1.** Binding energy score for fluoroquinolone analogues **FQH 1-5** with DNA topo II						
Bacterial	Reference Ligand (kcal/mol)	**FQH-1** (kcal/mol)	**FQH-2** (kcal/mol)	**FQH-3** (kcal/mol)	**FQH-4** (kcal/mol)	**FQH-5** (kcal/mol)
S. aureus (5cdq)	-11.30	-10.9	-10.6	-10.4	-8	-8.4
E. coli (6rkv)	-11.3	-10.9	-9.9	-9.2	-7.8	-8.9
K. pneumonia (5eix)	-10.6	-7	-9.1	-9.7	8.8	-7.1

FTIR – ATR spectra

A)

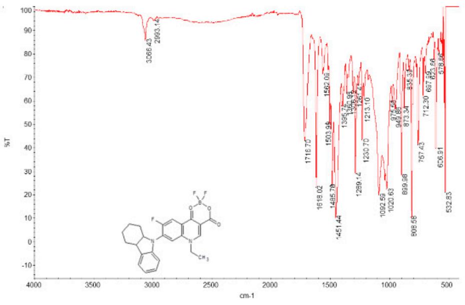

B)

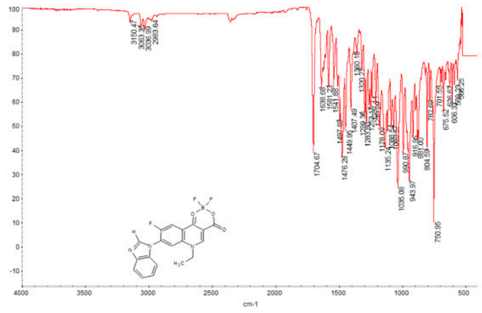

C)

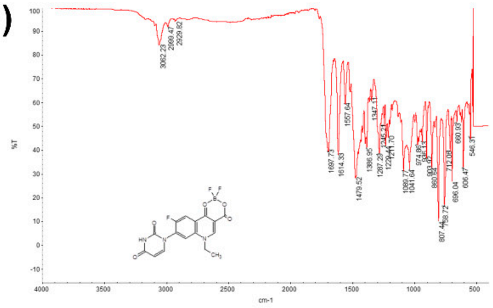

D)

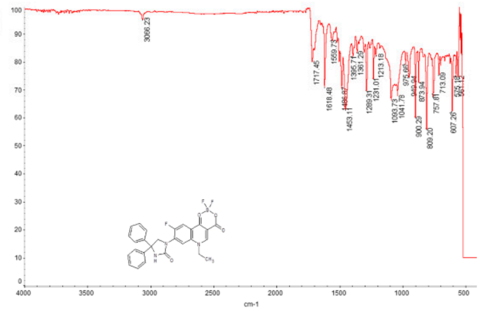

E)

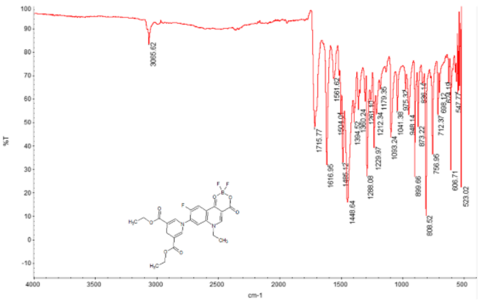

F)

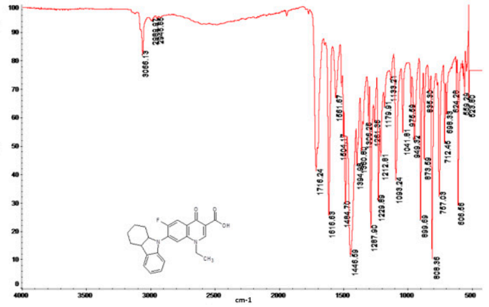

G)

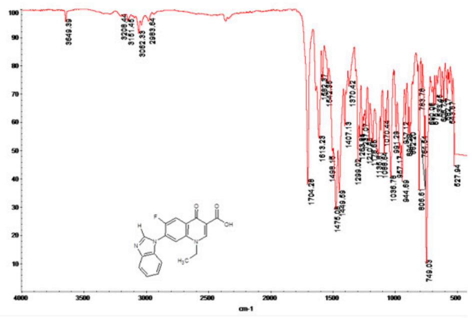

H)

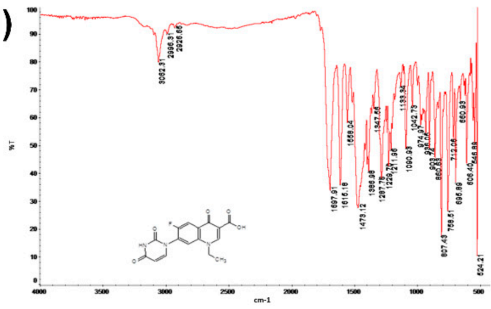

I)

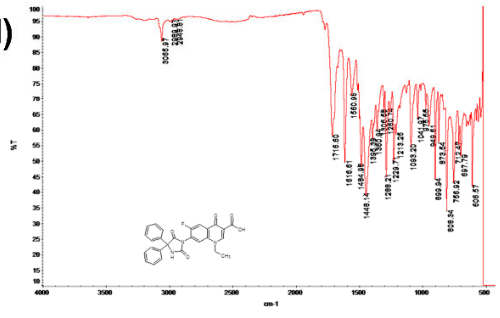

J)

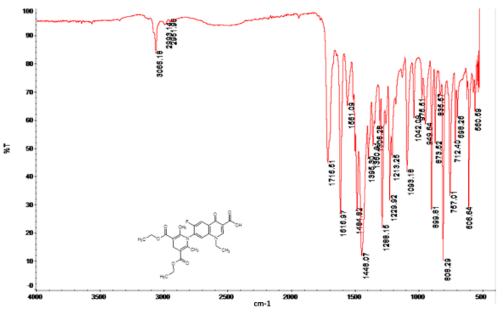

Figure S1. FTIR-ATR spectra of fluoroquinolone analogues FQB 1-5 and FQH 1-5. **A)** Difluoroboranyl 1-ethyl-7-(5*H*-1,2,3,4-tetrahydrocarbazol-5-yl)-6-fluoro-4-oxo-1,4-dihydroquinoline-3-carboxylate (**FQB-1**). **B)** Difluoroboranyl 1-ethyl-7-(1*H*-benzimidazol-1-yl)-6-fluoro-4-oxo-1,4-dihydroquinoline-3-carboxylate (**FQB-2**). **C)** Difluoroboranyl 1-ethyl-6-fluoro-4-oxo-7-(uracil-1-yl)-1,4-dihydroquinoline-3-carboxylate (**FQB-3**). **D)** Difluoroboranyl 1-ethyl-7-(5,5-diphenylhydantoin-3-yl)-6-fluoro-4-oxo-1,4-dihydroquinoline-3-carboxylate (**FQB-4**). **E)** Difluoroboranyl 1-ethyl-7-(3,5-diethoxycarbonyl-2,6-dimethyl-1,4-dihydropyridin-1-yl)-6-fluoro-4-oxo-1,4-dihydroquinoline-3-carboxylate (**FQB-5)**. **F)** 1-ethyl-7-(5*H*-1,2,3,4-tetrahydrocarbazole-5-yl)-6-fluoro-4-oxo-1,4-dihydroquinoline-3-carboxylic acid (**FQH-1**). **G)** 1-ethyl-7-(1*H*-benzimidazole-1-yl)-6-fluoro-4-oxo-1,4-dihydroquinoline-3-carboxylic acid (**FQH-1**). **H)** 1-ethyl-6-fluoro-4-oxo-7-(uracil-1-yl)-1,4-dihydroquinoline-3-carboxylic acid (**FQH-3**). **I)** 1-ethyl-6-fluoro-7-(5,5-diphenylhydantoin-3-yl)-4-oxo-1,4-dihydroquinoline-3-carboxylic acid (**FQH-4**). **J)** 1-(3-carboxy-1-ethyl-6-fluoro-4-oxo-1,4-dihydroquinolin-7-yl)-2,6-dimethyl-1,4-dihydropyridine-3,5-dicarboxylic acid (**FQH-5**).

.

NMR spectra

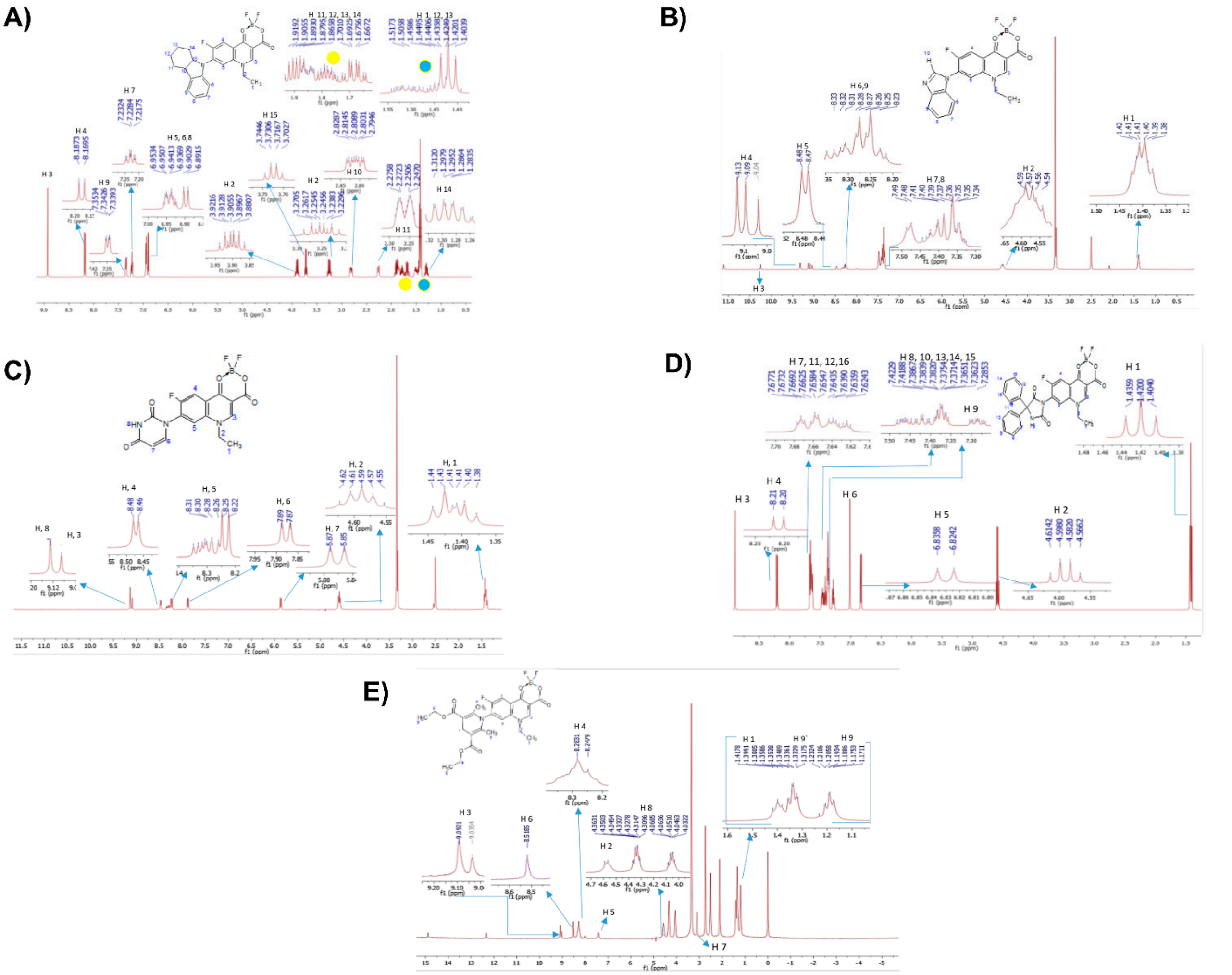

Figure S2. ^{1}H NMR of fluoroquinolone analogues FQB 1-5. A) Difluoroboranyl 1-ethyl-7-(5*H*-1,2,3,4-tetrahydrocarbazol-5-yl)-6-fluoro-4-oxo-1,4-difluoroboryl dihydroquinoline-3-carboxylate (**FQB-1**). **B)** Difluoroboranyl 1-ethyl-7-(1*H*-benzimidazol-1-yl)-6-fluoro-4-oxo-1,4-dihydroquinoline-3-carboxylate (**FQB-2**). **C)** Difluoroboranyl 1-ethyl-7-6-fluoro-4-oxo-7-(uracil-1-yl)-1,4-dihydroquinoline-3-carboxylate (**FQB-3**). **D)** Difluoroboranyl 1-ethyl-7-(5,5-diphenylhydantoin-3-yl)-6-fluoro-4-oxo-1,4-dihydroquinoline-3-carboxylate (**FQB-4**). **E)** Difluoroboranyl 1-ethyl-7-(3,5-diethoxycarbonyl-2,6-dimethyl-1,4-dihydropyridin-1-yl)-6-fluoro-4-oxo-1,4-dihydroquinoline-3-carboxylate (**FQB-5)**.

Thin Layer Chromatography

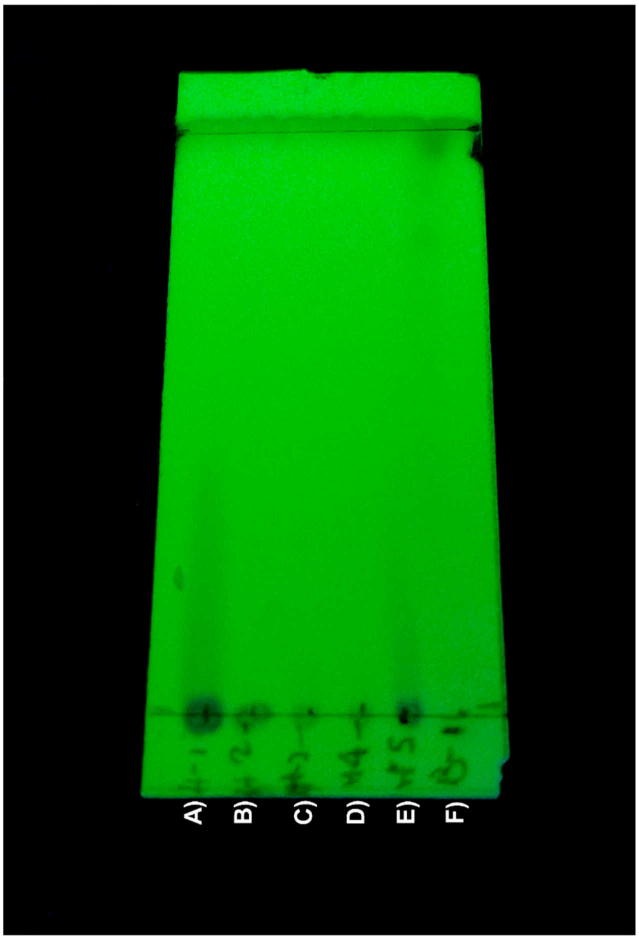

Figure S3. Thin Layer Chromatography (TLC) of fluoroquinolone analogues FQH 1-5. The TLC was made of **A) FQH-1** (7-(2,3,4,5-tetrahydro-carbazol-1-yl), **B) FQH-2** (7-benzimidazol-1-yl), **C) FQH-3** (7-uracil-1-yl), **D) FQH-4** (7-[5,5-diphenyl-hydantoin-1-yl]), **E) FQH-5** (7-[3,5-diethoxycarbonyl-2,6-dimethyl-1,4-dihydropyridin-yl]) and **F) FQB-1** (Difluoroboryl 7-(2,3,4,5-tetrahydro-carbazol-1-yl) in aqueous solution of distilled water and NaOH 0.25N (basic pH), using silica gel plate and a solution of acetonitrile/ethanol (95:5), as mobile phase.
