# Supplementary figures and images for: Fluoroquinolone Analogs, SAR Analysis, and the Antimicrobial Evaluation of 7-Benzimidazol-1-yl-fluoroquinolone in In Vitro, In Silico, and In Vivo Models
Source: Molecules. 2023 Aug 11;28(16):6018. doi: 10.3390/molecules28166018 (PMC10458221; doi:10.3390/molecules28166018)

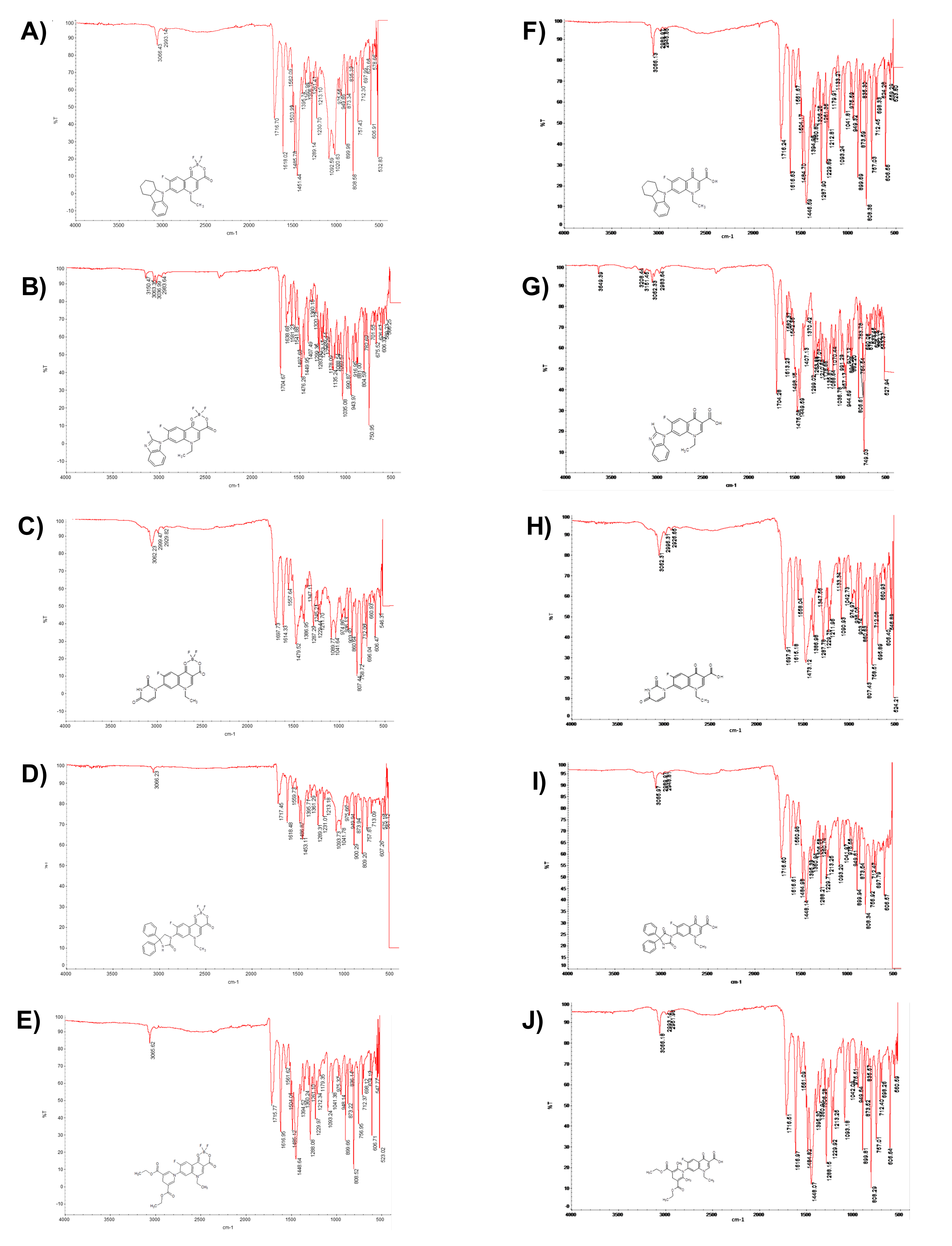

Supplement: Supplementary file 1 [file molecules-28-06018-s001.zip › Figure S1.tif]

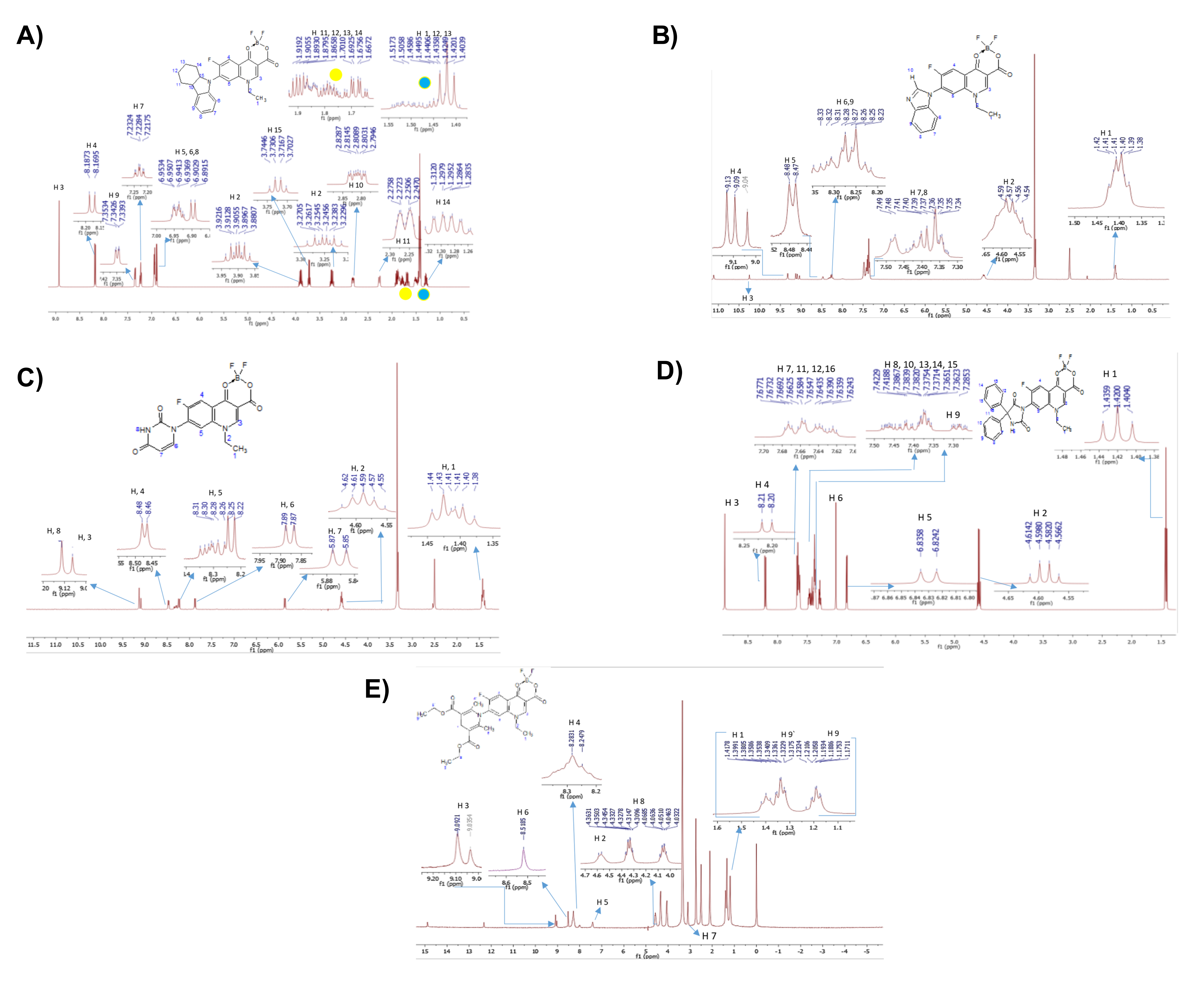

Supplement: Supplementary file 1 [file molecules-28-06018-s001.zip › Figure S2.tif]

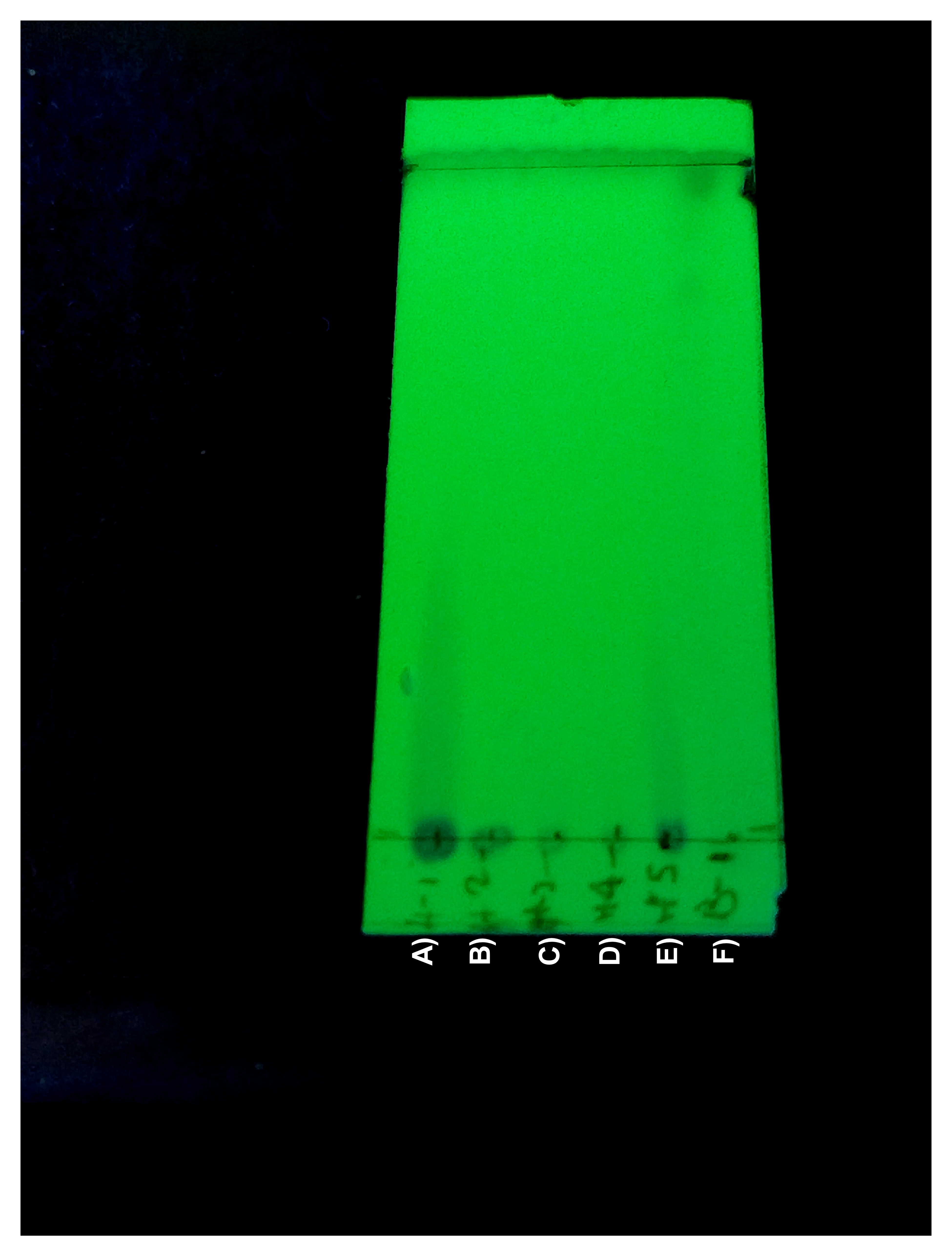

Supplement: Supplementary file 1 [file molecules-28-06018-s001.zip › Figure S3.tif]
